# Psychophysiological Adaptations to Exercise Training in COVID-19 Patients: A Systematic Review

**DOI:** 10.1155/2024/3325321

**Published:** 2024-05-02

**Authors:** Sameer Badri AL-Mhanna, Alexios Batrakoulis, Martin Hofmeister, Clemens Drenowatz, Wan Syaheedah Wan Ghazali, Georgian Badicu, Hafeez Abiola Afolabi, Mehmet Gülü, Yusuf Wada, Monira I. Aldhahi, Pantelis T. Nikolaidis

**Affiliations:** ^1^Center for Global Health Research, Saveetha Medical College and Hospitals, Saveetha Institute of Medical and Technical Sciences, Saveetha University, Chennai, Tamil Nadu, India; ^2^Department of Physiology, School of Medical Sciences, Universiti Sains Malaysia, Kubang Kerian, Kelantan, Malaysia; ^3^Department of Physical Education and Sport Science, School of Physical Education, Sport Science and Dietetics, University of Thessaly, Karies, Trikala, Greece; ^4^Department Food and Nutrition, Consumer Centre of the German Federal State of Bavaria, Munich, Germany; ^5^Division of Sport, Physical Activity and Health, University of Teacher Education Upper Austria, Linz, Austria; ^6^Department of Physical Education and Special Motricity, Transilvania University of Brasov, Brasov, Romania; ^7^Department of General Surgery, School of Medical Sciences, Universiti Sains Malaysia, Kubang Kerian, Kelantan, Malaysia; ^8^Department of Sports Management, Faculty of Sport Sciences, Kirikkale University, Kirikkale, Türkiye; ^9^Department of Zoology, Ahmadu Bello University, Zaria, Nigeria; ^10^Department of Rehabilitation Sciences, College of Health and Rehabilitation Sciences, Princess Nourah Bint Abdulrahman University, Riyadh, Saudi Arabia; ^11^School of Health and Caring Sciences, University of West Attica, Athens, Greece

## Abstract

**Introduction:**

Many COVID-19 patients display adverse symptoms, such as reduced physical ability, poor quality of life, and impaired pulmonary function. Therefore, this systematic review is aimed at evaluating the effectiveness of physical exercise on various psychophysiological indicators among COVID-19 patients who may be at any stage of their illness (i.e., critically ill, hospitalized, postdischarge, and recovering).

**Methods:**

A systematic search was conducted in PubMed, Scopus, ScienceDirect, Web of Science, and Google Scholar from 2019 to 2021. Twenty-seven studies, which assessed a total of 1525 patients, were included and analysed.

**Results:**

Overall, data revealed significant improvements in the following parameters: physical function, dyspnoea, pulmonary function, quality of life (QOL), lower limb endurance and strength, anxiety, depression, physical activity level, muscle strength, oxygen saturation, fatigue, C-reactive protein (CRP), interleukin 6 (IL-6), tumour necrosis factor-alpha (TNF-*α*), lymphocyte, leukocytes, and a fibrin degradation product (D-dimer).

**Conclusions:**

Physical training turns out to be an effective therapy that minimises the severity of COVID-19 in the intervention group compared to the standard treatment. Therefore, physical training could be incorporated into conventional treatment of COVID-19 patients. More randomized controlled studies with follow-up evaluations are required to evaluate the long-term advantages of physical training. Future research is essential to establish the optimal exercise intensity level and assess the musculoskeletal fitness of recovered COVID-19 patients. This trial is registered with CRD42021283087.

## 1. Introduction

The coronavirus disease 19 (COVID-19) is a novel infectious illness that has caused dramatic health effects and drastically impacted economic costs worldwide [1]. On March 11, 2020, the WHO officially declared the COVID-19 pandemic [2]. COVID-19 has had a massive impact and a high death rate worldwide [3]. To date, COVID-19 has caused 269 million cases and 5.3 million deaths across 224 countries and territories. Since the advent of the Omicron variant, daily case numbers have surged to 0.6 million with thousands of fatalities [4, 5]. Furthermore, those who recovered remain partly affected by the virus. Barriers to participation in more physical activity have increased dramatically with COVID-19 [6, 7]. Patients with severe COVID-19 have significant daily activity impairments and require multimodal rehabilitation with cardiovascular and pulmonary medicine expertise [8]. The residual impact of hospitalization length of stay and side effects of medication among recovered patients resulted in a decline in pulmonary function and cardiorespiratory deconditioning [9, 10]. Furthermore, a high prevalence of anxiety and depression has been documented post-treatment as well as anxiety in families [11].

The COVID-19 pandemic highlights the need for postacute care in individuals with a severe disease progression. It has been reported that after being released from the acute care unit, COVID-19 patients may not be able to return to their pre-COVID-19 functional state or baseline levels of healthcare need [12]. Long-term consequences are anticipated, and rehabilitation medicine is challenged in recovering physical performance and improving cognitive dysfunction [13]. However, much evidence indicates that COVID-19 is causing long-term effects for the survivors [14] and postacute syndrome manifested as shortness of breath, cognitive disturbances, fatigue, chest pain, and decreased quality of life [15, 16]. Thus, immobility syndrome and physical function impairment are common in post-COVID-19 patients, even in those who are only mildly affected [17]. However, early rehabilitation, such as mobilization, pulmonary rehabilitation, and therapeutic training, may positively impact patient recovery following COVID-19, especially in severe cases or those at risk of developing postintensive care syndrome [18]. Despite this, the need for postacute care, particularly rehabilitation after severe and catastrophic COVID-19 infections, is mandatory, yet it challenges the global healthcare systems [19, 20].

Although survivors with severe COVID-19 have chronic weakness and cardiorespiratory failure, the availability and potential benefit of cardiopulmonary rehabilitation and physical exercise after COVID-19 are unknown [21]. Moreover, in the literature, there is insufficient evidence of the effect of exercise on fitness determinants, inflammatory markers, physical function, strength, body composition, and sleep quality [22]. Interestingly, postrehabilitation exercise programs for people affected by COVID-19 have been discussed in the global health and fitness industry [23–25]. However, most data on the benefits of rehabilitation and physical activity as long-term care in postacute patients are unclear. The development and evaluation of effective rehabilitation programs are urgently needed [13, 26]. Therefore, this systematic review is aimed at determining the effectiveness of physical training among COVID-19 patients on fitness determinants, pulmonary function, and quality of life.

## 2. Materials and Methods

### 2.1. Protocol and Registration

The protocol was registered in the international database PROSPERO with the registration number CRD42021283087. Before the registration in PROSPERO, we carried out a formal screening of searches in PubMed, Scopus, ScienceDirect, Google Scholar, and Web of Science to check whether there were adequate studies related to our study or to ensure that no other systematic reviews on the same topic had been conducted, which is a requirement before the registration in PROSPERO.

### 2.2. Research Question and Outcome Measures

The study is aimed at determining the effectiveness of physical activity on the quality of life and physical function in COVID-19 patients. The study adhered to the preferred reporting items for systematic review and meta-analysis protocols (PRISMA-P 2016). Patients were selected based on the “PICOS” (participants, interventions, comparisons, outcomes, study design) process with the criteria as follows: P (population) = COVID-19 patients; I (intervention) = physical training; C (comparison) = control; O (outcome) = improvement in physical function, QOL, CRP, IL6, and pulmonary function; and S (study design) = clinical studies.

#### 2.2.1. Types of Outcome Measures

*(1) The Primary Outcomes*. The primary outcome included physical function (walking distance and muscle strength), dyspnoea, and pulmonary function among critically ill, hospitalized, postdischarge, and recovering COVID-19 patients.

*(2) The Secondary Outcomes*. Secondary outcomes included quality of life (QOL), lower limb endurance and strength, anxiety, depression, physical activity intensity level, oxygen saturation, fatigue, C-reactive protein (CRP), interleukin 6 (IL-6), tumour necrosis factor-alpha (TNF-*α*), lymphocyte, leukocytes, and D-dimer.

### 2.3. Data Sources and Literature Retrieval Strategy

Five databases, including PubMed, Scopus, ScienceDirect, Web of Science, and Google Scholar, were searched. Three independent authors (S.B.A., B.O.K., and H.A.) conducted an electronic literature search using keywords combined with the Boolean operations OR and “AND” to find relevant literature (Table 1). The search strategy involved a combination of subject terms and free words and was finalized after repeated checks. The keywords were (“physical activity∗” OR “exercise” OR “pulmonary rehabilitation” OR “telerehabilitation” OR “Respiratory rehabilitation” OR “training” OR “fitness”) AND (“Covid-19” OR “SARS-CoV-2” OR “2019-nCoV”).

#### 2.3.1. Eligibility Criteria

A literature search was carried out to identify experiments that investigated the impact of exercise on COVID-19 patients published between 2019 and 2021. Three authors (S.B.A., Y.W., and H.A.) used the PICOS strategy to examine the extensive texts of the remaining papers and define the inclusion and exclusion criteria. The judgment of a fourth author (A.B.) was employed to settle disagreements.

#### 2.3.2. Inclusion and Exclusion Criteria

This study involved COVID-19 patients with varying severity levels, including mild, moderate, and severe/critical cases. The study also considers the presence or absence of comorbidities and other underlying diseases, with no age limit, publications with no language limitation and with full text available, any structured physical exercise (aerobic training, resistance training, combined aerobic and resistance training, physical activity, or pulmonary rehabilitation exercise), and any relevant clinical experimental studies (RCT, pre-post study design, and non-RCT). Exclusion criteria were case reports, review articles, letters, commentaries, short communications, and studies with unclear data.

#### 2.3.3. Study Selection

Three authors, A.A.I., A.B., and H.A., evaluated the selection and exclusion of articles based on a linear assessment of names, abstracts, and full texts (in cases of doubt). The remaining papers were evaluated entirely based on the qualifying criteria before making a final selection. This method was used independently, with the assistance of a fourth author, M.G., in the case of any conflicts or doubts.

#### 2.3.4. Data Extraction

After reading the full article, three authors (H.A., S.A.B., and M.G.) conducted independent sampling and data extraction from qualifying studies. Specifically, information on the first author, journal name, population, year of publication, gender, and type of intervention (exercise mode, duration, intensity, sets, repetition, and exercise duration), study duration, and outcome measures was extracted.

#### 2.3.5. Assessment of Risk of Bias

**Figure 1:**
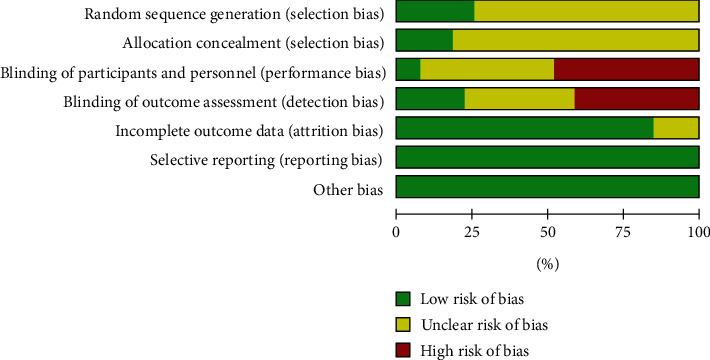
Risk of bias graph: review authors' judgments about each risk of bias item presented as percentages across all included studies (https://www.cochranelibrary.com/cdsr/doi/10.1002/14651858.CD005179.pub3/full).

**Figure 2:**
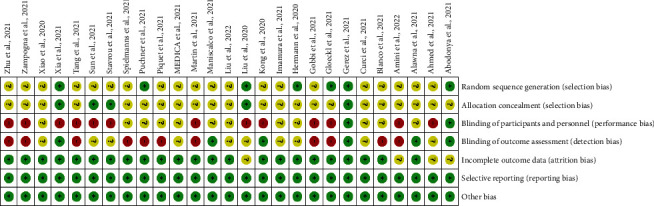
Risk of bias summary: review authors' judgments about each risk of bias item for each included study (https://www.cochranelibrary.com/cdsr/doi/10.1002/14651858.CD005179.pub3/full).

The assessment of the risk of bias was previously described [27]. In summary, we checked the risk of bias based on the Cochrane Handbook for Systematic Reviews of Interventions (Figures 1 and 2) [28].

### 2.4. Summary of Findings

The Cochrane Collaboration's Grades of Recommendation, Assessment, Development, and Evaluation (GRADE) approach was used to assess the quality of evidence of the included studies. The GRADE system provides four levels of quality, with randomized trial evidence being the highest. It might be degraded to moderate, low, or even extremely poor-quality evidence [29] (Table 2).

## 3. Results

### 3.1. Study Selection Results

**Figure 3:**
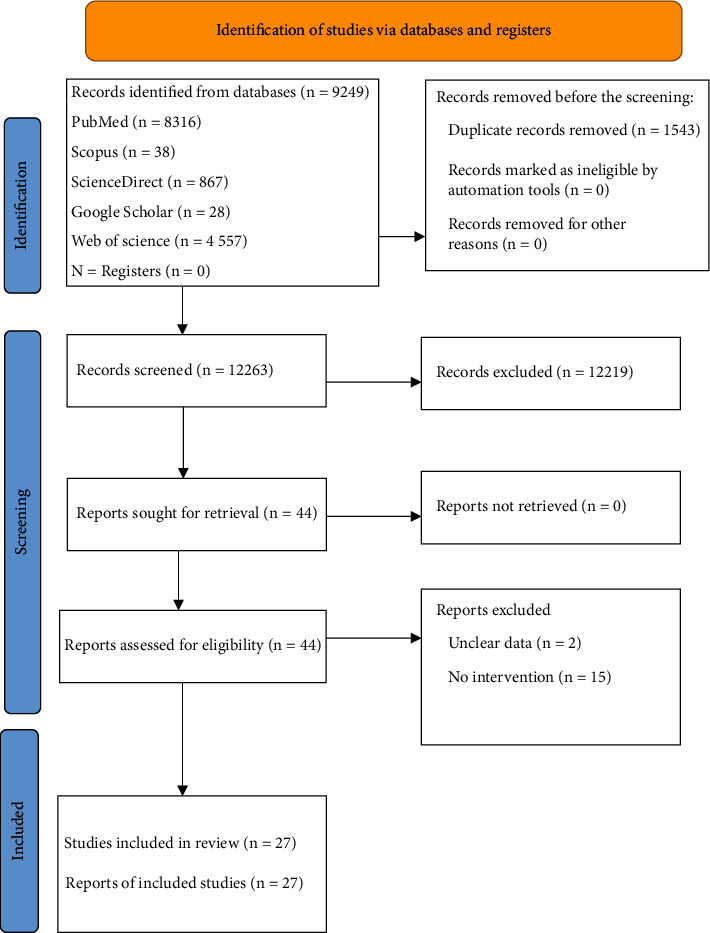
PRISMA flowchart for search strategy.

Figure 3 depicts the consort diagram of the study in which a total of 13,806 studies were retrieved. After identifying duplicate articles, 12,263 studies were screened for further selection. After reading the article's title and abstract, a total of 12,219 were excluded according to the inclusion and exclusion criteria. Thus, 44 articles proceeded for further selection by reading the full texts, out of which 17 were excluded. The remaining 27 articles that met the eligibility criteria were used for data extraction.

### 3.2. Study Features

Table 3 summarizes the major features of the studies included in this systematic review. The studies selected were carried out in different countries during various time durations. Each of the included studies was published in a good, reputed journal. The technical characteristics were population, type of exercise, duration of the training, intensity, sets, repetition, the timing of the intervention, duration of the activity, and outcome measures.

In the included studies, a total of 1525 patients were clinically assessed. The duration of the training was approximately one to eight weeks. In these studies, COVID-19 patients were introduced to the combination of various exercises like aerobic exercise (AE) [30]; motor training [31]; Liuzijue exercise (breathing exercise) [32]; telerehabilitation program [33]; a combination of AE, resistance training (RT), and breathing exercise (cardiopulmonary rehabilitation) [21]; rehabilitation program consisting of passive or active range of motion exercises [34]; respiratory rehabilitation programs [12, 13, 22, 26, 35–49]; and AE and RT [50, 51].

### 3.3. Outcome Measures

#### 3.3.1. Primary Outcomes

*(1) Walking Distance*. A significant improvement in the physical function of patients undergoing respiratory rehabilitation [13, 22, 26, 35–38, 40, 42–45, 47–49], AE and RT [50], and following Liuzijue exercise [32] was reported. Lack of significant differences was reported in physical function after AE and RT [51], after respiratory rehabilitation [12], and following AE, strength training, and breathing exercises [21] in COVID-19 patients.

*(2) Muscle Strength*. There were significant improvements in physical activity intensity levels following the respiratory rehabilitation [43, 51], while no significant difference following the Rb program consisting of passive or active range of motion exercises was observed [34].

*(3) Dyspnoea*. There was a significant improvement in dyspnoea in COVID-19 patients following respiratory rehabilitation [12, 26, 36, 38, 46, 47], while no significant differences in dyspnoea postrespiratory rehabilitation were observed [37, 42].

*(4) Pulmonary Function*. There was a significant improvement in pulmonary function following respiratory rehabilitation [13, 37, 40, 48, 49] and following Liuzijue exercise [32]. At the same time, few studies reported no significant difference in pulmonary function following respiratory rehabilitation [12, 22, 35, 36, 47].

#### 3.3.2. Secondary Outcomes

*(1) QOL*. There was a significant improvement in QOL following respiratory rehabilitation [22, 35, 36, 39–41, 43, 47] and following the Liuzijue exercise [32, 46]. No significant difference in QOL was reported following the respiratory rehabilitation in COVID-19 patients [37] and following the Rb program consisting of passive or active range of motion exercises [34] and progressive muscular relaxation training [33].

*(2) Peripheral Muscle Performance of Lower Limb*. There were significant improvements in the peripheral muscle performance of the lower limb following the respiratory rehabilitation [22, 38, 44, 47], while one study reported no significant improvements following the respiratory rehabilitation [48].

*(3) Anxiety*. There were significant improvements in anxiety following respiratory Rb [39–41], after motor training [31], and following progressive muscular relaxation training [33].

*(4) Depression*. There were significant improvements in depression following respiratory Rb [39, 40], after motor training [31], and following progressive muscular relaxation training [33].

*(5) Physical Activity Intensity Level*. There were significant improvements in physical activity levels following the respiratory rehabilitation [38, 44].

*(6) Oxygen Saturation*. There were significant improvements in oxygen saturation following the respiratory rehabilitation [22, 26], while no significant difference was observed following the respiratory Rb [37] and AE, strength training, and breathing exercise [21].

*(7) Fatigue*. There were significant improvements in fatigue following the respiratory rehabilitation [12, 26].

*(8) CRP, IL6, and TNF-α*. There was a significant improvement in CRP following respiratory rehabilitation [26, 46] and AE and RT [50]. At the same time, few studies reported no significant difference in CRP [37, 49], following respiratory Rb, and following passive or active range of motion exercises [34]. One study reported a significant reduction in IL6 and TNF after AE [30].

*(9) Lymphocyte*. There was a significant improvement in lymphocytes following respiratory rehabilitation [46] and AE [30]. There was no significant difference in lymphocytes following AE and RT [50], passive or active range of motion exercises [34], and respiratory rehabilitation [49].

*(10) Leukocytes*. There was a significant improvement in leukocytes following AE [30], while there was no significant difference following the respiratory rehabilitation [37].

*(11) D-Dimer*. There was a significant improvement in D-dimer following respiratory rehabilitation [37] and AE and RT [50], while no significant difference in D-dimer was observed following passive or active range of motion exercises [34], as well as respiratory rehabilitation [49].

### 3.4. Quality of the Evidence

The quality of trial evidence was mainly very low, and only a few had moderate certainty. There was a low or unclear risk of bias for most trials in most domains, and there is no evidence of selective reporting bias. In the original research and subsequent review, a lack of proper random sequence generation could lead to treatment effect bias in the original trial and the subsequent review. Very low to moderate heterogeneity was found in the studies, indicating that the overall quality of the evidence supporting this review, as determined by the GRADE method, ranges from very low to moderate quality.

### 3.5. Potential Biases in the Review Process

For additional details, we searched many databases without language restrictions and checked all the reference lists to identify all the relevant studies. We cannot declare with absolute certainty, however, that we have identified all the articles in this field. All selected papers met all criteria for inclusion, and no bias was introduced throughout the review process. Secondary citations were verified, and each study underwent a thorough examination.

## 4. Discussion

This study is aimed at determining the effectiveness of physical exercise among COVID-19 patients. The obtained review findings proved to be interesting in terms of public health, considering the impact of the epidemic period on COVID-19 patients. To the best of our knowledge, this is the first systematic review that investigated the effects of different types of physical exercise on COVID-19 patients. However, prior research examined the impacts of pulmonary rehabilitation using various study methodologies but did not encompass diverse forms of exercise intervention [52–55].

We observed significant improvements in the following parameters: physical function including walking distance and muscle strength, dyspnoea, pulmonary function, QOL, peripheral muscle performance of lower limb, anxiety, depression, physical activity level, oxygen saturation, fatigue, CRP, IL-6, TNF-*α*, lymphocyte, leukocytes, and D-dimer. These findings aligned with the previous studies [53, 54, 56–58]. Interestingly, Cuenca-Zaldivar et al. [56] delved into the impacts of four physiotherapy interventions, namely, strength exercises, seated exercises, cardiovascular exercises, and balance and walking exercises. Their research revealed notable enhancements in physical fitness and reductions in frailty through the implementation of multicomponent exercise programs.

In the included RCTs, the results revealed significant differences in health outcomes within the intervention group. However, there was no significant difference in the control group. This could be attributed to the beneficial effects of exercise on immune function, respiratory health, and overall well-being, which might have been more pronounced among those actively engaging in physical activity.

However, the current review included different types of exercise, and some of them showed no significant improvement in physical function following AE and RT [51], respiratory rehabilitation [12], and AE, strength training, and breathing exercises [21] in COVID-19 patients. The use of different measures in the studies, the multivariate nature of QOL, exercise type, duration and intensity, a lack of high-quality research, or differences in study populations might all contribute to the insignificant improvement of these measures.

Despite a considerable improvement in exercise performance, mild/moderate COVID-19 patients were released with a degraded 6-minute walk distance (81% pred). In some reports, even a year after the COVID-19 acute phase, it has been shown that the 6-minute walking distance test may be markedly below typical reference values [59]. However, mild to severe COVID-19 patients in the same trial increased the 6-minute walking distance by 48 m, significantly above the recommended minimal significant difference of 30 m in patients with respiratory illnesses (88% of patients surpassed this threshold) [60]. Despite that, the patient was in a six-month post-SARS-CoV-2 infection phase, and a significant increase in six-minute walking distance within three weeks of pulmonary rehabilitation was reported. This suggests that the impact of pulmonary rehabilitation cannot be completely ruled out. It seems that referring COVID-19 patients to pulmonary rehabilitation following the illness's acute phase may enhance exercise capacity recovery. Despite this significant improvement, patients with severe/critical COVID-19 still only attained 70.5% of their anticipated 6MWD after pulmonary rehabilitation. Considering that patients' quadriceps strength returned to normal (99.6% pred) at the end of pulmonary rehabilitation, this could be connected to the enduring respiratory capacity limitations rather than to skeletal muscle weakness [37].

Most of our included studies showed significant improvement in dyspnoea, but few reported no significant difference in dyspnoea postrespiratory rehabilitation [37, 42]. The exact mechanisms underlying this low impact of respiratory rehabilitation should be investigated explicitly in dedicated studies. The low effect of respiratory rehabilitation might be due to the severity and long-term impact of COVID-19 infection or usual symptoms for far longer than expected [61]. The duration of the intervention should also be considered when conducting respiratory rehabilitation among COVID-19 patients. Therefore, one of our included studies reported no significant difference in dyspnoea after four weeks of intervention, but the follow-up of six weeks showed significant improvements in dyspnoea postrespiratory rehabilitation in COVID-19 patients [47]. A previous study reported significant improvement in dyspnoea, respiratory function, quality of life, and anxiety among the patients who participated in the study rehabilitation program [57].

Three months after the beginning of symptoms, 33% of COVID-19-hospitalized patients exhibit aberrant patient-reported outcome measures, with 33% reporting at least substantial impairments in critical quality domains [62]. However, most of our included studies showed significant improvement in QOL. At the same time, few reported no significant difference in QOL following respiratory rehabilitation in COVID-19 patients [37], following a passive or active range of motion exercise rehabilitation program [34], and following a telerehabilitation program of muscular relaxation training [33]. This might be due to the use of various quality-of-life scales and the variances in the type, duration, and intensity of exercise, a scarcity of high-quality research, or discrepancies in study populations [63, 64]. Usually, COVID-19 patients have psychological distress [16]. This review showed significant improvement in anxiety and depression following physical training. Therefore, we primarily attributed these improvements to the effects of physical training, including targeted interventions concentrating on disease management and coping with COVID-19 and its aftereffects.

Some of our included studies reported no significant difference in pulmonary function following respiratory rehabilitation [12, 22, 35, 36, 47]. A previous study assessed the impact of rehabilitation on 29 studies involving COVID-19 patients and showed a decrease in the severity and progression of COVID-19-related disorders and an improvement in pulmonary function and quality of life [65].

The most relevant limitation of our study is the inclusion of only a few randomized controlled trials. Some of the included studies consisted of COVID-19 patients without a control group due to ethical issues of withholding a known effective treatment. Another limitation of the current study was the heterogeneity of included studies, which included different types of physical training. Apart from this, there was no such limitation for the review process.

## 5. Conclusions

### 5.1. Implications for Practice

Our study showed that exercise training is a practical, feasible, and safe way to improve physical function, dyspnoea, pulmonary function, QOL, peripheral muscle performance of lower limbs, anxiety, depression, physical activity intensity level, muscle strength, oxygen saturation, fatigue, CRP, IL-6, TNF-*α*, lymphocytes, leukocytes, and D-dimer in COVID-19 patients. Therefore, promoting physical training early in the stage of recovery is recommended to mitigate the negative consequences of the disease. The results of this review could be considered in the context of clinical findings. Additional studies, however, are suggested to enhance the understanding of the appropriate training parameters and exercise prescriptions to enhance the physical and psychological condition of patients recovering from COVID-19.

### 5.2. Implications for Research

More randomized controlled studies with follow-up evaluations are required to evaluate the long-term advantages of physical training. Future research is essential to establish the proper exercise intensity level and to assess the musculoskeletal fitness of recovered COVID-19 patients. Additionally, research on COVID-19 and other respiratory illnesses should be compiled concerning aerobic and resistance training. Finally, robust evidence based on high-quality trials and effort is required to support nonpharmaceutical intervention to enhance the health and well-being of individuals recovered from COVID-19 [66].

## Figures and Tables

**Table 1:** Search strategy.

1. PubMed	(“physical activity∗”[Title/Abstract] OR “exercise” [Title/Abstract] OR “rehabilitation” [Title/Abstract] OR “telerehabilitation” [Title/Abstract] OR “training” [Title/Abstract] OR“fitness” [Title/Abstract]) AND (“Covid-19” [Title/Abstract] OR “SARS-CoV-2” [Title/Abstract] OR “2019-nCoV” [Title/Abstract]).
2. Scopus	TITLE- ABS (“Physical activity∗” OR “exercise” OR “rehabilitation” OR “telerehabilitation” OR “training”, “fitness”) AND TITLE- ABS (“Covid-19” OR “SARS-CoV-2” OR “2019-nCoV”).
3. ScienceDirect	(“Physical activity” OR “exercise” OR “rehabilitation” OR “telerehabilitation” OR “training”, “fitness”) (“Covid-19” OR “SARS-CoV-2” OR “2019-nCoV”).
4. Google Scholar	Allintitle: (“Physical activity” OR “exercise” OR “rehabilitation” OR “telerehabilitation” OR “training” OR “fitness”) (“Covid-19” OR “SARS-CoV-2” OR “2019-nCoV”)
5. Web of science	(“Physical activity” OR “exercise” OR “rehabilitation” OR “telerehabilitation” OR “training” OR “fitness”) (“Covid-19” OR “SARS-CoV-2” OR “2019-nCoV”)

**Table 2:** Summary of finding using GRADE quality assessment.

Outcome	Certainty assessment							No. of patients		Certainty
	No. of studies	Study design	Risk of bias	Inconsistency	Indirectness	Imprecision	Other considerations	Intervention group	Control group	
Physical function	20	3 APS 5 RCT 2 PCS 3 RS 5 OS 2^∗^	Serious^c^	Very serious^b^	Not serious	Serious^a^	None	945	228	⨁◯◯◯ Very low
Dyspnoea		APS = 2 RCT = 2 RS = 1 OS = 2 1^∗^	Serious^c^	Very serious^b^	Not serious	Serious^a^	None	320	93	⨁◯◯◯ Very low
Pulmonary function		APS = 1 RCT = 2 PCS = 1 RS = 1 OS = 2 2^∗^	Serious^c^	Very serious^b^	Not serious	Serious^a^	None	551	178	⨁◯◯◯ Very low
Quality of life		APS = 3 RCT = 4 PCS = 1 RS = 1 OS = 3 1^∗^	Serious^c^	Very serious^b^	Not serious	Serious^a^	None	501	258	⨁◯◯◯ Very low
Peripheral muscle performance of lower limp		RCT = 3 RS = 1 1^∗^	Serious^c^	Very serious^b^	Not serious	Serious^a^	None	247	98	⨁◯◯◯ Very low
Anxiety	5	QM = 1 RCT = 3 OS = 1 1^∗^	Serious^c^	Very serious^b^	Not serious	Serious^a^	None	200	159	⨁◯◯◯ Very low
Depression	4	QM = 1 RCT = 2 OS = 1	Serious^c^	Very serious^b^	Not serious	Serious^a^	None	130	89	⨁◯◯◯ Very low
Physical activity intensity level	2	RCT = 2	Not serious	Not serious	Not serious	Serious^a^	None	37	37	⨁⨁⨁◯ Moderate
Muscle strength	3	RS = 2 OS = 1	Serious^d^	Very serious^b^	Not serious	Serious^a^	None	145	17	⨁◯◯◯ Very low
Oxygen saturation	4	RS = 1 OS = 1 2^∗^	Serious^c^	Very serious^b^	Not serious	Serious^a^	None	139	—	⨁◯◯◯ Very low
Fatigue	2	OS = 1 1^∗^	Not serious	Very serious^b^	Not serious	Serious^a^	None	136	—	⨁◯◯◯ Very low
CRP	6	APS = 1 OS = 5	Serious^c^	Very serious^b^	Not serious	Serious^a^	None	216	17	⨁◯◯◯ Very low
Interleukin-6 and tumour necrosis factor-alpha	1	RCT = 1	Not serious	Not serious	Not serious	Serious^a^	None	15	15	⨁⨁⨁◯ Moderate
Lymphocyte	5	APS = 1 RCT = 1 OS = 3	Serious^c^	Very serious^b^	Not serious	Serious^a^	None	175	92	⨁◯◯◯ Very low
Leukocytes	2	RST = 1 OS = 1	Serious^c^	Very serious^b^	Not serious	Serious^a^	None	65	15	⨁◯◯◯ Very low
D-dimer	4	OS = 4	Serious^c^	Very serious^b^	Not serious	Serious^a^	None	179	77	⨁◯◯◯ Very low

^a^The included studies recorded a small sample size for both the control and intervention groups. ^b^There is considerable heterogeneity in the studies. ^c^Participants and assessors were not blinded. ^d^The assessors were not blinded. ^∗^Not stated. APS = a prospective interventional study; RCT = randomized control trial; PCS = pilot control clinical study; RS = retrospective study; OS = observational study; QM = a quasiexperimental repeated measure.

**Table 3:** Characteristics of the included studies.

Reference	Population and sample Size	Participants/age	Gender	Intervention (type of exercise, duration, intensity, sets, and reps.)	Time of intervention & study duration	Study design	Parameter/the outcome measure	PRO measure instrument	Results	Conclusion
Ahmed et al. [36]	Pakistan (*N* = 20)	COVID-19 patients Age: 38.0 ± 10.3 years old	Male and female	All participants underwent (3 S/WK) of AE (20–60 M/S) and breathing exercises for (10 M/S)	In postdischarge COVID-19 patients 5 weeks	A prospective interventional study	(1) HRQOL (2) Breathlessness (3) Cardiorespiratory fitness (4) Pulmonary function	(1) SF-36 (2) Modified Borg Dyspnoea Scale (3) 6MWT test (4) FVCs	Significant improvement in the following: (1) Dyspnoea: pre-EX: 14.2 ± 3.5 and post-EX: 17.1 ± 4.8 (*p* = 0.001) (2) Physical function: pre: 332.6 ± 34.5 and post: 376.5 ± 39.4 (*p* ≤ 0.001) (3) QOL: pre: 38.6 ± 5.8 and post: 59.4 ± 8.3 (*p* ≤ 0.001) (4) No significant result for pulmonary function: pre: 78.7 ± 13.5 and post: 84.2 ± 10.3 (*p* = 0.047)	In COVID-19-recovered individuals, cardiorespiratory fitness and health-related QOL were substantially improved. As a result, an early Rb training program should be implemented in COVID-19-recovered patients to increase cardiorespiratory fitness and QOL. Greater improvement in general health and body pain domains of health-related QOL
Xia et al. [47]	China (*N* = 120) EX (*n* = 59) CO (*n* = 61)	Hospitalized COVID-19 survivors with remaining dyspnoea Age: 49.17 ± 10.75 years old	Male and female	Participants were subjected to 6 WK of telerehabilitation program (unsupervised home program) 3–4 S/WK. Included: breathing control and thoracic expansion, AE, and lower limb muscle strength EX	In postdischarge COVID-19 patients 6 weeks	RCT	(1) Physical function (2) Peripheral muscle performance of lower limp (3) Pulmonary function (4) QOL (5) Dyspnoea	(1) 6MWT (2) Squat time in seconds (3) Spirometry (4) SF-12 (5) —	Significant improvement in the following: (1) Physical function: pre: 80.20 ± 74.66 and post 84.81 ± 80.38 (*p* ≤ 0.001) (2) Peripheral muscle performance of lower limp: pre: 28.12 ± 27.17 and post: 29.35 ± 27.22 (*p* ≤ 0.001) (3) QOL: pre: 39.15 ± 7.16 and post: 7.81 ± 7.02 (*p* = 0.04) (4) Dyspnoea: pre: 1.27 (0.88 to 1.82), after 4 WK: 1.08 (0.82 to 1.42) (*p* = 0.605), and after 6 WK: 1.46 (1.17 to 1.82) (*p* = 0.001) (5) No significant different in pulmonary function: pre: 0.21 ± 0.47 and post: 0.30 ± 0.38 (*p* = 0.95) However, there was no significant difference in the control group (*p* > 0.05)	Self-reported dyspnoea and maximal voluntary breathing had short-term effects. Otherwise, the intervention's benefits on pulmonary function are doubtful, and its impacts on the mental elements of QOL are minimal. No serious adverse events were recorded
Liu et al. [40]	China (*N* = 72) EX (*n* = 36) CO (*n* = 36)	Elderly patients with COVID-19 Comorbidities (HTN, T2DM, and osteoporosis) Age: 69.4 ± 8.0 years old	Male	Participants were subjected to respiratory Rb of 2 S/WK for 10 M. The intervention includes respiratory muscle EX, cough EX, diaphragmatic EX, stretching EX, and home EX at 60% of the individual's maximal expiratory mouth pressure	With a definite diagnosis of COVID-19 6 weeks	RCT	(1) PFT (2) Functional tests (3) QOL (4) Mental status tests	(1) DLCO (2) 6MWT (3) SF-36 (4) SAS anxiety and SDS depression scores	Significant improvements the following: (1) Pulmonary function: pre: 1.79 ± 0.53 and post: 2.36 ± 0.49 (2) Physical function: pre: 162.7 ± 72.0 and post: 212.3 ± 82.5 (3) QOL: pre: 52.4 ± 6.2 and post: 71.6 ± 7.6 (4) Decreased SAS: pre: 56.3 ± 8.1 and post: 47.4 ± 6.3 (5) SDS scores: pre: 56.4 ± 7.9 and post: 54.5 ± 5.938 (*p* ≤ 0.05) However, there was no significant difference in the control group (*p* > 0.05)	RB program can improve QOL, respiratory function, anxiety, and depression of elderly patients with COVID-19
Kong et al. [39]	China (*N* = 16) EX (*n* = 13) CO (*n* = 13)	COVID-19 patients Age: 49.98 ± 13.73 years old	Male and female	Patients undergo breathing exercises for 20 M every D, the EX was based on Yoga breathing methods, and it aims to stimulate nasal and diaphragmatic breathing, increase expiratory duration, reduce respiratory flow, and regulate breathing rhythm	During diagnosed with COVID-19 10 days	RCT	(1) Depression and anxiety (2) QOL	(1) HADS-A and HADS-D (2) PSSS	Significant reduction in the following: (1) HADS scores of depressions (pre: 12.62 ± 2.663 and post: 6.15 ± 3.579) and anxiety (pre: 11.69 ± 2.926 and post: 5.92 ± 3.730) (2) QOL: pre: 54.69 ± 15.585 and post: 64.46 ± 11.050 (*p* ≤ 0.05) There was no significant difference in the control group (*p* > 0.05)	Breathing EX proper intervention and critical element for COVID-19 patients' treatment as well as having a beneficial effect on COVID-19 patients' mental health and better life quality
Spielmanns et al. [45]	Switzerland (*N* = 99)	COVID-19 patients Age: 67.72 ± 10.23 years old Reported comorbidities	Male and female	Pulmonary rehabilitation program including a total of 25–30/S for 5–6 WK/D. Endurance (cycling and treadmill) 5-6/WK for 10–30/M/S, gymnastics 5–6/WK for 45 M/S, outdoor walking 2–3/W for 45 M/S, strength training 3–4/WK for 30 M/S, relaxation 2/WK for 45 M/S, and respiratory therapy 3/for 30 M/S	Post-COVID-19 patients 3 weeks	Observational study	(1) Physical function	(1) 6 MWT	Significant improvements in the following: (1) Physical function: pre: 176 ± 141 and post: 357 ± 132 (*p* ≤ 0.05)	Pulmonary Rb has been linked to significant clinical and functional gains in those who have had severe COVID-19
Zampogna et al. [48]	Italy (*N* = 140)	COVID-19 patients Age: 71.0 (61.5–78.0) years old	Male and female	The EX (1 depends on the patient's ability) were mobilization, active exercises and free walking, peripheral limb muscle activities, shoulder, and full arm circling for 30 M/daily/S. The EX (2 depends on the patient's ability) was strengthening, callisthenic, cycling, balance EX, and paced walking	Recovered COVID-19 patients — Between April 1 and August 15, 2020. Short	Retrospective study	(1) Pulmonary function (2) Performance of lower extremity function (3) Exercise tolerance	(1) Barthel Index (2) SPPB (3) 6MWT	Significant improvement in the following: (1) Pulmonary function: 55.0 (30.0–90.0) to 95.0 (65.0–100.0) (*p* = 0.01) (2) SPPB: post: 0.5 (0–7) (mean ± SD = 3.2 ± 3.7) (3) Physical function: pre: 47.7 ± 18.9 and post: 68.4 ± 15.3 (*p* ≤ 0.05)	Pulmonary Rb is effective and successful in COVID-19 survivors, including those who require assisted breathing or oxygen management. It may also be valuable to guide physicians caring for COVID-19 survivors and enhance their quality of life
Abodonya et al. [35]	Saudi Arabia (*N* = 42) EX (*n* = 21) CO (*n* = 21)	COVID-19 patients Age: 48.05 ± 8.85 years old	Male and female	The IMT group was utilizing a threshold inspiratory muscle trainer at 2/S/D for 5/D/WK. Each S consisted of six inspiratory cycles, each lasting about 5 M of resisted inspiration followed by a 60-second rest period. The inspiratory threshold pressure was set at 50% of the maximum inspiratory pressure (MIP)	Recovered COVID-19 patients 2 weeks	Pilot control clinical study	(1) Pulmonary function test (FVC%, FEV1%) (2) Dyspnoea Severity Index (3) QOL (4) Functional capacity	(1) Spirometer (2) DSI questionnaires (3) Euro Quality-5 Dimensions-3 Levels (EQ-5D-3L) questionnaire (4) 6MWT	No significant difference in the following: (1) Pulmonary function: pre: 78.7 ± 13.5 and post: 84.2 ± 10.3 (*p* = 047) Significant improvements in the following: (2) DSI score: pre: 18.5 ± 4.3 and post: 14.2 ± 3.5 (*p* = 0.001) (3) QOL: pre: 38.6 ± 5.8 and post: 59.4 ± 8.3 (4) Physical function: pre: 332.6 ± 34.5 and post: 376.5 ± 39.4 (*p* ≤ 0.001)	After consecutive weaning from mechanical ventilation, a 2 WK of IMT improves pulmonary functions, dyspnoea, functional performance, and QOL in recovered ICU COVID-19 patients. In the COVID-19 treatment program, the IMT should be emphasized, especially with ICU patients
Amini et al. [31]	Iran (*N* = 42)	COVID-19 patients Age: 70.03 ± 5.42 years old	Elderly male	Motor training was standing on the support platform, walking between obstacles, striking the ball, and walking on a narrow support surface while holding an object. The cognitive training was a countdown, reverse spelling, and poem reading. The intensity and duration were 1 to 5 h dual-task training programs (motor and cognitive training). Each training S lasted an average of 45 M and included 6 EX in 2 to 3 sets (5–10 repetitions/set). Participants completed a 10-M/S. The EX was 2S/WK	Recovered from the COVID-19 4 weeks	A quasiexperimental repeated measure	(1) Anxiety and depression	(1) GHQ-2	Significant improvement in the scores of anxiety (pre: 13.777 ± 2.448 and post: 11.733 ± 2.348) and depression (pre: 8.589 ± 1.660 and post: 7.580 ± 1.431)	This study contributes to the existing knowledge about the usefulness of cognitive-motor training in regaining cognitive health in older individuals who have recovered from COVID-19, and it corroborates cognitive-motor training as a feasible therapeutic strategy
Curci et al. [26]	Italy (*N* = 41)	COVID-19 patients Age: 72.15 ± 11.07 years old Reported comorbidities	Male and female	The Rb program included passive mobilization, posture changes, clapping and vibration, breathing EX, with diaphragm recruitment and chest-abdomen coordination EX; passive muscle stretching and pumping EX. All patients underwent Rb intervention (30 M/set, 2/D) Patients completed balancing and coordination EX such as one-legged stance, static heel/toes, and walking for escalating distances at the end of the Rb program	Postacute COVID-19 patients 31.97 ± 9.06 D	Real-practice retrospective study	(1) Disability status (2) Dyspnoea in activities of daily living (3) Physical function (4) Effort and exertion, breathlessness, and fatigue during physical work (5) Oxygen saturation (6) CRP	(1) Barthel Index (2) Dyspnoea Scale (mMRC) (3) 6MWT (4) RPE (5) SpO_2_ (6) Serum levels of laboratory markers	Significant improvement in the following: (1) B1: pre: 43.37 ± 26.00 and post: 84.87 ± 15.56 (*p* ≤ 0.001) (2) Dyspnoea: pre: 37 ± 90.2 and post: 0 ± 0.0 (*p* ≤ 0.001) (3) Physical function: pre: 240.0 ± 81.31 and post: 303.37 ± 112.18 (*p* = 0.028) (4) Fatigue: pre: 16.03 ± 2.28 and post: 12.23 ± 2.51 (*p* ≤ 0.01) (5) Oxygen saturation: pre: 94.66 ± 2.65 and post: 95.43 ± 1.46 (*p* = 0.088) (6) Reduced in the levels of CRP: pre: 0.77 ± 1.19 and post: 6.57 ± 6.94 (*p* ≤ 0.01)	Postacute COVID-19 patients can benefit from the motor and respiratory Rb therapy
Gloeckl et al. [37]	Germany (*N* = 50)	Mild/moderate and severe/critical COVID-19 patients Age: 60–71 years old	Male and female	Pulmonary Rb which included cycle endurance training for 10–20 M/S at 60–70% of peak work rate 5D/WK, strength training which included leg press, knee extension, and other EX such as back extension and abdominal trainer three sets/EX at an individual intensity 15–20 repetitions. RT for 30 M/S 5D/WK, patient education 2 S/WK, respiratory physiotherapy 2-4 times/WK for 30 M, activities of daily living training 4-5/WK for 30 M	A mild to critical COVID-19 patients 3 weeks	Prospective, observational cohort study	(1) Physical function (2) Pulmonary function (3) QOL (4) Dyspnoea (5) Laboratory parameters (6) Oxygen saturation (7) Leukocytes (8) D-dimer	(1) 6MWT (2) FVC (3) SF-36 (4) Borg scale (5) (6) Handgrip strength kg peak quadriceps strength % pred and five-rep STST (7) SPO_2_	Significant improvement in the following: (1) Physical function: pre: 509 (426–539) and post: 557 (463–633) (*p* = 0.009) (2) Pulmonary function: pre: 80.0 (59.2–90.9) and post: 87.7 (67.0–98.9) (*p* ≤ 0.001) (3) QOL: no significant difference; in severe COVID-19 patients: pre: 30.2 (22.7–36.8) and post: 34.7 (30.2–41.3) (*p* = 0.59) (4) Dyspnoea: pre: 5 (4–6) and post: 5 (3–6) (*p* = 0.83) (5) CRP: pre: 2.6 (1.5–5.4) and post: 2.0 (1.3–3.9) (*p* = 0.95) (6) Oxygen saturation: pre: 92.0 (87.8–94.2) and post: 93.0 (85.5–94.5) (*p* = 0.19) (7) There were no significant improvements in leukocytes: pre: 7.2 (6.0–9.7) and post: 7.0 (6.0–9.7) (*p* = 0.19) (8) D-dimer: pre: 726 (367–982) and post: 428 (307–807) (*p* = 0.01)	Pulmonary Rb is practicable and with a high rate of adherence, safe (no adverse events), and helpful in improving EX performance, lung function, and quality of life in patients with mild/moderate and severe/critical COVID-19
Gobbi et al. [50]	Italy (*N* = 48)	COVID-19 patients Age: 68.7 ± 11.8 years old	Male and female	Rb program includes EX for body conditioning such as sit to stand, simple bed EX, limb muscle strengthening (8-12 reps, 1-3 sets with 2 M rest between sets), and AE with cycle and arm ergometer for 45 M/S for 5D/W at moderate intensity (65%VO2M)	After discharge from acute COVID-19	Observational study	(1) Physical function (2) Laboratory findings	(1) Timed-up and-go (2) Serum blood	(1) Physical function: pre: 25.4 ± 19.5 and post: 16.3 ± 15.9 (*p* ≤ 0.001) Significant reduction in the following: (2) CRP: pre: 1.6 ± 1.5 and post: 0.9 ± 1.1 (*p* = 0.015) (3) Lymphocyte: pre: 2.2 ± 0.9 and post: 2.3 ± 0.9 (*p* = 0.257) (4) D-dimer: pre: 1435.8 (1441.9) and post: 776.6 (778.0) (*p* = 0.017)	In post-SARS-CoV-2 patients, a dietary intervention aerobic and strengthening EX improved functional status, CRP, and D-dimer
Gonzalez-Gerez et al. [38]	Spain (*N* = 38) EX (*n* = 19) CO (*n* = 19)	Mild to moderate symptomatology in the acute-stage COVID-19 patients Age: 40.79 ± 9.84 years old	Male and female	Pulmonary Rb program, the breathing EX was 1/D for 7 D at home; based on the assessment of the RPE, patients underwent 4 (RPE 7–10) for 10 M), 8 (RPE 5–7) for 20 M), or 12 (RPE <5) reps/EX/D for 30 M)	In confined COVID-19 patients in the acute phase 1 week	Randomized, controlled, parallel, double-blind, two-arm	(1) Physical function (2) Peripheral muscle performance of lower limbs (3) Multidimensional nature of dyspnoea (4) Physical activity intensity level	(1) 6MWT (2) Thirty-second STST (3) Multidimensional dyspnoea-12 (4) RPE	Significant improvement in the following: (1) Physical function: pre: 374.72 ± 151.59 and post: 487.58 ± 133.36 (*p* = 0.006) (2) Peripheral muscle performance of lower limbs: pre: 12.68 ± 5.33 and post: 14.00 ± 5.47 (*p* = 0.001) (3) Dyspnoea: pre: 12.68 ± 5.33 and post: 14.00 ± 5.47 (*p* = 0.001) (4) Physical activity intensity level: pre: 5.58 ± 2.32 and post: 2.95 ± 1.27 (*p* ≤ 0.001) There was no significant difference in the control group (*p* > 0.05)	Breathing EX by telerehabilitation was a viable method for improving physical condition, dyspnoea, and perceived effort in persons with mild to severe COVID-19 symptoms in the acute phases, suggesting therapeutic benefits, compliance, and a safe approach
Imamura et al. [51]	Brazil (*N* = 27)	COVID-19 patients Age: 53.78 ± 13.34 years old	Male and female	Rb program included stretching, muscle strengthening, mobilization, functional, and RT, including active cycle ergometer 2-3 times/W K using RPE to monitor intensity, activities for the lower limbs; functional electrical stimulation-assisted training; sensory stimulation; orthostatic positioning; balance, gait, and body awareness training; and safety guidance for performing activities of daily living independently	After recovered from COVID-19 22.70 ± 9.49 D	Retrospective study	(1) Muscle strength (2) Physical function	(1) Medical Research Council sum score (2) The short physical performance battery (SPPB)	Significant improvement in the following: (1) Muscle strength: pre: 43 81 ± 7.76 and post: 50.67 ± 7.45 (*p* ≤ 0.001) (2) No significant difference in physical function: pre: 5.92 ± 1.19 and post: 8.75 ± 2.11	Rb improves patients' functional status following COVID-19 recovery and should be addressed in postacute COVID-19 patients
Liu et al. [41]	China (*N* = 140) EX (*n* = 70) CO (*n* = 70)	Patients with mild COVID-19 infections Age: 28 (40.00%)	Male and female	Pulmonary RB, included five-tone breathing EX (five-step breathing EX and two-section motion EX, combining a five-element music therapy). A set of Baduanjin EX for 1 D is 30 M, and a course of treatment lasts for 7 D	During COVID-19 infections	RCT	(1) Anxiety state (2) QOL	(1) SAI (2) PQSI	Significant improvement in the following: (1) SAI: 38.5 ± 13.2 (*p* = 0.01) (2) QOL: 5.6 ± 3.0 (*p* = 0.003) However, there was no significant difference in the control group (*p* > 0.05)	Pulmonary Rb had a significant effect on anxiety and sleep disorders in patients with mild COVID-19 infections
Maniscalco et al. [12]	Italy (*N* = 95)	COVID-19 patients Age: 65.3 ± 1.2 years old Reported comorbidities	Male and female	All patients performed pulmonary Rb program for 6 S/WK, including physical EX training at moderate to high intensities (strengthening muscles in the upper and lower extremities, treadmill walking and cycling)	After discharge 5 Weeks		(1) Pulmonary function (2) Physical function (3) Dyspnoea (4) Fatigue (5) 6MWT was also performed in accordance with the ATS/ERS guidelines (6) Dyspnoea and fatigue	(1) Single-breath method (2) Automated equipment (Vyasis, Milan, Italy, according to American Thoracic Society/European Respiratory Society (ATS/ERS) guidelines) (3) — (4) — (5) — (6) —	No significant improvements in the following: (1) Respiratory function: pre: 326.3 mL (95% CI: -130.6–783.3) and post 430.9 mL (95% CI: -20.6–882.5) (*p* = 0.06) (2) Physical function: pre: 42.0 m (95% CI: -1.9–85.8) and post: 39.7 m (95% CI: -2.5–81) (*p* = 0.06) (3) Dyspnoea score was reduced to 0.4 (95% CI: 0.03–0.8 (*p* = 0.03) (4) Reduced fatigue 0.6 (95% CI: 0.1–1.1) (*p* = 0.04)	The multidisciplinary Rb program is highly beneficial in post-COVID-19 patients, regardless of the underlying cardiorespiratory comorbidity. No adverse events were recorded
Martin et al. [42]	Belgium (*N* = 27) EX (*n* = 14) CO (*n* = 13)	Severe or critical COVID-19 patients Age: 60.8 ± 10.4 years old	Male and female	The pulmonary Rb was carried out at home 2/WK. Each S comprised 30 M of endurance EX and upper and lower body muscle strengthening. The RPE score was used to determine the intensity of the endurance exercise. The upper and lower body muscular training was done using items found in the participants' homes (bottles of water and a chair). For each exercise, the participants were told to perform 2–3 series of 8–12 reps	Patients hospitalized with COVID-19 6 weeks	Prospective observational study	(1) Functional exercise capacity (2) Dyspnoea (3) HR and SPO_2_	(1) STST (2) Visual analog scale (3) Finger pulse oximeter	No significant different in the following: (1) physical function: 17.6 ± 4.7 (15.1; 20.0) (*p* < 0.001) (2) Dyspnoea: 0 (0–3), 2 (0–5) (*p* = 0.836) (3) SPO_2_: 91.8 ± 3.3 (90.0; 93.5) (*p* = 0.568)	Patients in the hospital with COVID-19 had limited functional discharge ability and poor recovery after three months. The feasibility and efficacy of a basic telerehabilitation program have been confirmed, and the functional recovery after three months has significantly improved
Medica, Edizioni Minerva [34]	Turkey (*N* = 35) EX (*n* = 18) CO (*n* = 17)	Acute respiratory distress syndrome patients with COVID-19 Age: 64–78 years old Reported comorbidities	Male and female	Rb program includes a passive range of motion EX being carried out. Each joint in the extremities was moved passively for 10-15 reps, for a total of 15 M/D, for 6 D/WK	During hospitalization Admission median 1-14 D	Observational study	(1) Muscle strength (2) HRQOL (3) Laboratory findings	(1) Medical Research Council (MRC) Scale (2) SF-36 (3) Serum blood	No significant different in the following: (1) Muscle strength: 58 (50–60), 48–60 (*p* = 1.000) (2) QOL: 90 (38–100) (*p* = 0.580) (3) CRP: 280 (146–331), 68–412 (*p* = 0.869) (4) IL 6: 138 (71–2593), 28–20 276 (*p* = 0.449) (5) Lymphocyte: 0.40 (0.20–0.53), 0.00–0.90 (*p* = 0.854) (6) IL6: 138 (71–2593), 28–20,276 (*p* = 0.449) (7) D-dimer: 8515 (2735–10,108), 1420–13,666 (*p* = 0.234) (8) Ferritin: 280 (146–331), 68–412 (*p* = 0.869)	The findings did not support the idea that early intensive care unit Rb improves muscular strength. More individuals in the rehab group with pulmonary and neurologic disorders may reduce the effect of Rb on outcomes. These comorbidities, on the other hand, highlight the need for Rb. When appropriate measures are followed, it is safe for both patients and healthcare professionals
Piquet et al. [43]	France (*N* = 100)	COVID-19 patients with 49% had hypertension, 29% had diabetes, and 26% had more than 50% pulmonary damage on computed tomographic scans Age: 66 ± 22 years old	Male and female	2 S/D was given for each patient as part of the Rb program but was short (<20 M). The program consisted of body weight EX (sit-to-stand, tiptoe stands, squats), elastics, and weights, with each EX consisting of 3 series of 10 reps. Respiratory Rb was associated with controlled diaphragmatic breathing. AE comprised submaximal intensity cycling ergometer sessions	Patients hospitalized with COVID-19 9.8 ± 5.6	Retrospective chart review	(1) Activities of daily living (2) Physical function (3) Muscle strength	(1) Barthel activities of daily living index (2) — (3) Dynamometry	Significant improvement in the following: (1) QOL: pre: 77.3 ± 26.7 and post: 88.8 ± 24.5 (*p* < 0.001) (2) Physical function: pre: 0.27 ± 0.16 and post: 0.37 ± 0.16 (*p* < 0.001) (3) Muscle strength: pre: 18.1 ± 9.2 and post: 20.9 ± 8.9 (*p* < 0.001)	Inpatient Rb for COVID-19 patients was linked with significant motor, respiratory, and functional improvement, particularly in severe instances, but modest chronic autonomy loss persisted after discharge. COVID-19, mainly a respiratory illness, may progress to a motor disability as time in critical care increases
Puchner et al. [13]	Austria (*N* = 23)	COVID-19 patients Age: 57 ± 10 years old Reported comorbidities	Male	Breathing therapy, individual respiratory muscle training, mobilization and breathing perception therapy, endurance and strength training, speech therapy and swallow evaluation, occupational therapy, neuropsychological therapy, nutritional counselling, and passive therapy session (massages) training were conducted in S of 25 to 50 M	COVID-19 survivors 3 weeks	Observational cohort study.	(1) Pulmonary function (2) Physical function	(1) FVC (2) 6MWT	Significant improvements (1) Pulmonary function: pre: 3.0 ± 0.8 and post: 3.3 ± 0.7 (*p* = 0.007) (2) Physical function: pre: 323 ± 196 and post: 499 ± 103 (*p* ≤ 0.001)	Individuals discharged from the hospital after a severe COVID-19 infection often have persistent physical and cognitive dysfunctions. Multidisciplinary inpatient Rb is significantly effective for these individuals
Rodriguez-Blanco et al. [44]	Spain (*N* = 36) EX (*n* = 18) CO (*n* = 18)	COVID-19 patients with mild to moderate symptomatology Age: 39.39 + 11.7 years old		Telerehabilitation program with nonspecific conditioning EX included 10 EX based on nonspecific toning EX of resistance and strength, 1 S/D for 7 D, depending on RPE, at home. Patients completed 4 (RPE 7_10), 8 (RPE 5_7), or 12 (RPE 15) reps/EX/D for 10, 20, and 30 M, respectively. Patients also received a text message every D	Confined patients affected by COVID-19 in the acute phase 1 week	A randomized, controlled, parallel, double-blind, two-arm clinical trial	(1) Physical function (2) Peripheral muscle performance of lower limbs (3) Physical activity intensity level	(1) 6MWT (2) 30STST (3) RPE	Significant improvements in the following: (1) Physical function: pre: 414.56 ± 184.86 and post: 492.00 ± 183.44 (*p* = 0.016) (2) Peripheral muscle performance of lower limbs: pre: 12.78 ± 6.63 and post: 15.22 ± 7.51 (*p* = 0.011) (3) Physical activity intensity level: pre: 4.78 ± 1.99 and post: 2.22 ± 0.83 (*p* ≤ 0.001) However, there was no significant difference in the control group (*p* > 0.05)	90% adherence was found in the program In COVID-19 patients with mild to moderate symptoms in the acute stage, a 1 WK telerehabilitation program based on respiratory EX is effective, safe, and practical
Stavrou et al. [22]	Greece (*N* = 20)	COVID-19 survivor, being smokers (10%) and with COPD (10%), hypertension (65%), diabetes mellitus (20%), and CVD (10) Age: 64.1 ± 9.9 years old	Male and female	Unsupervised pulmonary Rb program; each patient participated in 3 S/WK. Each training S lasted about 100 M. Each training S comprised (i) flexibility and mobility EX warm-up and 5 M, (ii) recover set (5 M), (iii) the AE set with walking (50 M), (iv) the set with yoga EX for breathing and/or proprioception (20 M), and (v) the set with multijoint strength M (20 M). The patients in the AE set walked on a level, hard surface, and every 5 M patients measured their HR and SPO_2_	After recovery 8 weeks		(1) Pulmonary function (2) QOL (3) Physical function (4) Peripheral muscle performance of lower limbs (5) Oxygen saturation (6) Dyspnoea	(1) FVC (2) PSQI (3) 6MWT (4) 30 STST (5) Nonin 9590 Onyx Vantage, USA (6) Borg Scale CR10	No significant Improvement in the following: (1) Pulmonary function: pre: 84.8 ± 15.7 and post: 88.6 ± 14.7 (*p* = 0.214) (2) Significant improvements in QOL: pre: 0.6 ± 1.0 and post: 0.2 ± 0.7 (*p* = 0.031) (2) Physical function: pre: 94.6 ± 2.9 and post: 95.8 ± 3.2 (*p* = 0.013) (3) Peripheral muscle performance of lower limbs: pre: 11.4 ± 3.2 and post: 14.1 ± 2.7 (*p* ≤ 0.001) (4) Oxygen saturation: pre: 1.7 ± 1.3 and post: 1.5 ± 2.3 (*p* = 0.025) (5) Dyspnoea: pre: 1.3 ± 1.5 and post: 0.6 ± 0.9 (*p* = 0.005)	The research results recommend using unsupervised pulmonary Rb programs in patients who have recovered from COVID-19, to improve several aspects of long-term COVID-19 syndrome
Tang et al. [32]	China (*N* = 33)	Mild/moderate severe/critical COVID-19 patients 44.8 ± 11.0 years old	Male and female	Liuzijue EX routine was 1/D for 20 M	After discharge survivor over 4 WK	Multicentre prospective self-controlled s	(1) Pulmonary function (2) Physical function (3) QOL	(1) POWERbreathe inspiratory muscle assessment system (POWERbreathe International Ltd., UK) (2) 6MWT (3) SF36	Significant improvements in the following: (1) Pulmonary function: post: 0.74 ± 0.58 (*p* ≤ 0.001) (2) Physical function: post: 17.22 ± 43.78 (*p* = 0.020) (3) QOL: *p* = 0.009	Liuzijue EX is a safe and effective home exercise program that improves functional capacity and quality of life in COVID-19 patients who were discharged. These results also demonstrated the need for Rb for COVID-19 individuals who have been cured
Xiao et al. [33]	China (*N* = 79) EX (*n* = 39) CO (*n* = 40)	COVID-19 patients 58.45 ± 11.08 years old	Male and female	Patients were given progressive muscular relaxation training in bed for 30 M before waking up and 30 M before going to bed. The following information was provided: to begin, exert muscular tension and focus on the sensation of tension; attempt to maintain this sensation of tension for 3 to 5 seconds, then relax for 10 to 15 seconds. Following that, the patient should feel a sense of muscular relaxation. Patients were then taught how to relax in the following order: foot, leg, hip and waist, chest, arm, shoulder, and face training. Each S lasted 15 M for 1 WK	During isolation treatment 1 WK	A clinical observational study	(1) Anxiety status of patients (2) Depression status (3) QOL	(1) GAD-7 (2) PHQ-9 (3) PSQI	Significant difference in the following: (1) Anxiety: pre: 5.38 ± 5.25 and post: 3.69 ± 2.99 (*p* = 0.008) (2) Depression: pre: 5.05 ± 4.86 and post: 3.69 ± 3.93 (*p* = 0.003) (3) QOL: pre: 10.25 ± 2.75 and post: 7.41 ± 2.42 (*p* = 0.012)	In isolation therapy, progressive muscle relaxation training in COVID-19 patients can significantly decrease anxiety and sadness and enhance sleep quality. Advanced muscle relaxation training has been proven to improve patient care and is worthy of clinical support
Zhu et al. [49]	China (*N* = 123) EX (*n* = 63) CO (*n* = 60)	Patients with COVID-19 36.59 ± 7.01 years old	Male and female	Pulmonary Rb includes the following EX: (1) allowing patients to maintain regular mobility in the isolation ward, such as chest expansion and ambulation, for at least 1 hour/D; (2) providing respiratory control training; (3) purse-lip breathing: allow the patients to breathe deeply through their nose, hold their breath for 2 seconds, and then breathe deeply from their abdomen for 3–5 seconds with their mouth pursed as if whistling; the patients were taught for 10–15 M each and 4 times/D	During hospitalization between February 1, 2020, to March 31, 2020	A prospective observational study	(1) Physical function (2) Pulmonary function (3) Laboratory findings	(1) 6MWT (2) Spirometry (3) Serum blood	(1) Physical function: pre: 462.12 ± 31.61, post (one week of intervention): 495.88 ± 34.67 (*p* < 0.05) (2) Pulmonary function: pre: 2.05 ± 0.26, post (one week of intervention): 2.11 ± 0.29 and after (24 W of interventions): 2.95 ± 0.15 (*p* ≤ 0.05) (3) CRP: 23 ± 34 (*p* = 0.34) (4) D-dimer: pre: 1.7 ± 2.4 (*p* = 0.24) (5) White blood cell: 7.14 ± 3.41 (*p* = 0.63) (6) Lymphocyte: 0.62 ± 0.08 (*p* = 0.20)	Pulmonary Rb may accelerate the improvement of pulmonary function in COVID-19 patients
Hermann et al. [21]	Switzerland (*N* = 28)	Reported comorbidities; 66 years old	Male and female	The patients underwent a multimodal 2 to 4 WK inpatient cardiopulmonary Rb. This program was for 25–30 therapy S, for 5–6 D/WK, including AE and strength training. The intensity for AE was determined by a 6 MW. Strength training was done 3 times for a total of 20 reps	After severe COVID-19 2 to 4 WK A mean duration of 20 D		(1) Physical function (2) Oxygen saturation	(1) 6MWT (2) SPO_2_	No significant improvement in the following: (1) Physical function: 341.6 ± 134.7 (*p* = 0.391) (2) Oxygen saturation: 96.1 ± 2.1 (*p* = 0.946)	Following COVID-19, complete cardiopulmonary Rb is safe, practical, and successful. Physical performance and subjective health condition improved independently of previous ventilation
Sun et al. [46]	China (*N* = 31)	Patients with COVID-19 60.39 ± 10.20 years old	Male and female	Pulmonary Rb EX included (I) breathing exercises, (II) respiratory muscle training, (III) stretching training, and (IV) psychotherapy. Patients can perform pulmonary Rb (2 S/D for 3 WK) in the isolation	Inpatients confirmed COVID-19. 3 weeks	A self-pre- and postcontrol prospective clinical trial	(1) Dyspnoea (2) CRP (3) QOL (4) Laboratory findings	(1) Modified Medical Research Council (mMRC) dyspnoea scale (2) — (3) Using activity of daily living score (4) Serum blood	Significant improvements in the following: (1) CRP (*p* ≤ 0.0) (2) Dyspnoea (*p* = 0.03) (3) QOL (*p* ≤ 0.01) (4) Lymphocyte: pre: 1.16 ± 0.67 and post: 1.64 ± 0.57 (*p* ≤ 0.01)	In severe COVID-19 patients, PR can relieve symptoms, improve health-related quality of life, improve respiratory muscle function, and reduce disease-related anxiety and stress. PR should be given throughout the primary health management process, whether the patient is in the hospital or at home
Mohamed and Alawna [30]	Turkey (*N* = 30) EX (*n* = 15) CO (*n* = 15)	Mild or moderate COVID-19 patients 44.56 ± 4.25 years old	Male and female	Moderate-intensity AE for 40/S, 3/S/WK for 30 M, including treadmill walking/running or stationary bicycle riding. A 5 M warm-up slow walking or biking is followed by a 5 M cooldown EX (walking/running or bicycling) for each S. The EX intensity was 60-75% of the maximal HR estimated (MHR = 210—age). The exercise intensity was controlled using the RPE scale	COVID-19 patients during home quarantine 2 weeks	Observational study	(1) IL6 and TNF-*α* (2) Leucocytes and lymphocyte	(1) ELISA commercial kits assay (R&D Systems, Minneapolis, USA) (2) Lymphocytes and leukocytes from total-blood samples utilizing a multichannel hemocyte analysis system (SE-9000; Sysmex Corp, Hyogo, Japan)	Significant improvements in the following: (1) IL-6: pre: 23.05 ± 11.60 (*p* ≤ 0.01) (2) TNF-*α*: pre: 10.52 ± 0.78 and post: 11.25 ± 0.86 (*p* ≥ 0.05) (3) Leucocytes: pre: 5.44 ± 1.32 and post: 7.34 ± 1.40 (*p* ≤ 0.01) (4) Lymphocytes: pre: 0.98 ± 0.19 and post: 2.09 ± 0.18 (*p* ≤ 0.01)	2 WK of moderate-intensity AE reduced the severity and progress of COVID-19-related diseases, improved quality of life, and improved immunological function by elevating leucocytes, lymphocytes, and immunoglobulin A levels

## Data Availability

All data are available by the corresponding author upon reasonable request.
